# Germline Met Mutations in Mice Reveal Mutation- and Background-Associated Differences in Tumor Profiles

**DOI:** 10.1371/journal.pone.0013586

**Published:** 2010-10-25

**Authors:** Carrie R. Graveel, Jack D. DeGroot, Robert E. Sigler, George F. Vande Woude

**Affiliations:** 1 Department of Molecular Oncology, Van Andel Research Institute, Grand Rapids, Michigan, United States of America; 2 Research Essential Services, LLC, Plymouth, Michigan, United States of America; The University of Hong Kong, China

## Abstract

**Background:**

The receptor tyrosine kinase Met is involved in the progression and metastasis of numerous human cancers. Although overexpression and autocrine activation of the Met signaling pathway are commonly found in human cancers, mutational activation of Met has been observed in small cell and non-small cell lung cancers, lung adenocarcinomas, renal carcinomas, and mesotheliomas.

**Methodology/Principal Findings:**

To investigate the influence of mutationally activated Met in tumorigenesis, we utilized a novel mouse model. Previously, we observed that various Met mutations developed unique mutation-specific tumor spectra on a C57BL/6 background. Here, we assessed the effect of genetic background on the tumorigenic potential of mutationally activated Met. For this purpose, we created congenic knock-in lines of the Met mutations D1226N, M1248T, and Y1228C on the FVB/N background. Consistent with the mutation-specific tumor spectra, several of the mutations were associated with the same tumor types as observed on C57BL/6 background. However, on the FVB/N background most developed a high incidence of mammary carcinomas with diverse histopathologies.

**Conclusions/Significance:**

This study demonstrates that on two distinct mouse backgrounds, Met is able to initiate tumorigenesis in multiple cell types, including epithelial, hematopoietic, and endothelial. Furthermore, these observations emphasize that even a modest increase in Met activation can initiate tumorigenesis with both the Met mutational spectra and host background having profound influence on the type of tumor generated. Greater insight into the interaction of genetic modifiers and Met signaling will significantly enhance our ability to tailor combination therapies for Met-driven cancers.

## Introduction

Aberrant activity of tyrosine kinases through overexpression and/or mutation has been associated with numerous human cancers. The receptor tyrosine kinase ErbB2 (Her2/Neu) is overexpressed in 20% of breast cancers [1]; epidermal growth factor receptor (EGFR) is highly expressed in non-small-cell lung cancers [2]; and the FLT3 receptor is mutated in 30% of acute myelogenous leukemias (AML) and other hematological malignancies [3]. These studies and numerous others have demonstrated the significant impact of altered tyrosine kinase signaling in tumor initiation and progression in various tissue types. Those cancers that are addicted to the activation of a specific tyrosine kinase are optimal targets for therapy. Consequently, tyrosine kinases have been a substantial focus for the development of targeted therapeutics.

The receptor tyrosine kinase Met is an oncogene involved in the progression and metastasis of numerous human cancers [4]. The Met receptor is a disulfide-linked heterodimer containing an extracellular region (including a sema domain, a cysteine-rich domain, and four IgG domains), a transmembrane region, and an intracellular region (including the juxtamembrane domain, the kinase domain, and the C-terminal docking site). In normal physiological conditions, Met is expressed primarily by epithelial cells at the cell surface and is activated through paracrine binding of the ligand hepatocyte growth factor/scatter factor (HGF/SF) [5], [6], [7]. In neoplastic conditions, increased Met activity occurs through numerous mechanisms including overexpression of Met and/or HGF/SF, autocrine signaling, or mutational activation. In transformed cells, Met activation triggers several signaling cascades, such as the mitogen-activated protein kinase (MAPK) and AKT pathways, which result in proliferation, invasion, and/or prevention of apoptosis. Overexpression of Met and/or HGF/SF correlates with aggressiveness and poor outcome in most human cancers (www.vai.org/met) [4]. Since Met-HGF/SF signaling is involved in the progression of so many human cancers, it has the potential for being a significant therapeutic target. Currently, there are at least 12 Met and HGF/SF inhibitors in clinical trials [8]; these range from small molecule inhibitors to monoclonal antibodies tested against a wide spectrum of cancers.

Mutational activation of Met can induce tumor growth. Kinase domain mutations in Met are found in papillary renal carcinomas [9], [10]. This was the first observation to indicate that Met plays a role in tumor initiation in human cancer. More recently, Met mutations have been identified within the sema domain, juxtamembrane domain, and intronic regions in small cell and non-small cell lung cancers, lung adenocarcinomas, gastric cancer, renal carcinomas, and mesotheliomas [11], [12], [13], [14], [15]. Thus, mutational activation of Met is not restricted to renal cancers and may be a more common mechanism by which Met is aberrantly activated during tumorigenesis. Further studies have shown that several of these mutations induce resistance to several Met kinase inhibitors [16], [17], [18]. Therefore, additional studies are required to understand the effect of Met mutations in tumor progression and therapeutic resistance.

Mouse models have been essential for understanding the roles of tyrosine kinases in both normal mammalian development and tumorigenesis. To understand the role of Met in tumor development and progression, we have utilized a unique mouse model that allows us to compare four different mutations knocked into the *Met* genomic locus [19]. These Met mutants range from high to low in tyrosine kinase activity [20], [21], [22] and develop a unique tumor spectrum of primarily sarcomas and lymphomas on a C57BL/6;129/SV (B6) background [19]. Based on similar observations in other model systems showing that genetic background could have a profound influence on the tumor spectrum [23], we assessed the effect of these Met mutations in another genetic background.

We first evaluated the effect of the activating Met mutation M1248T/L1193V on the FVB/N (FVB) background [24]. M1248T and L1193V were first identified as somatic mutations in renal carcinomas and when combined as M1248T/L1193V have the highest kinase activity [9], [20], [22]. FVB mice carrying this mutation develop aggressive mammary carcinomas with varied histological features similar to basal-like breast cancers. This phenotype provided us with a unique opportunity to evaluate the role of Met in breast cancer. Further evaluation of human breast cancer tissues determined that MET overexpression was highly correlated with ER^–^/ERBB2^–^ and basal-like breast cancers [24]. To gain insight into how specific Met mutations induce tumorigenesis, we utilized the panel of knock-in mutations we previously generated. For this purpose we created congenic lines of the Met tyrosine kinase domain mutations M1248T, Y1228C, and D1226N, on the FVB background. Interestingly, except for D1226N, each mutation was associated with a distinct tumor spectrum compared to the identical mutation on the B6 background. Since the only differences between these animals are the Met mutations and the background, this study illustrates that either the kinase mutational structure itself or the level of kinase they impose (or both) influence the tissue-specificity for tumor formation. However, it also clearly demonstrates that mutation-specific tumor spectra are dramatically affected by the mouse background and sets the stage for future work aimed at defining which modifier loci genetically interact with Met mutations to influence the tumor spectrum.

## Results

### Assessing mutational activation of Met on the FVB background

Previously, we developed a knock-in mouse model of activating mutations in the Met receptor [19]. This model contains germline mutations in the endogenous *met* locus. For these studies, heterozygous animals were used due to the embryonic lethality that was observed in homozygous *Met^M1248T^*, *Met^Y1228C^*, and *Met^M1248T/L1193V^* mice. Some homozygous knock-in animals were also aged for the *Met^D1226N^* line. Each line carried one mutant allele expressed under the endogenous promoter; therefore phenotypic differences were not due to differences in insertion sites (as found in transgenic models).

Four mouse lines were initially generated on a B6 background and are referred to according to the effect the change has on the amino acid sequence of Met: *B6-Met^D1226N^*; *B6-Met^Y1228C^*; *B6-Met^M1248T^*; and *B6-Met^M1248T/L1193V^*. Previously, we found with the FVB strain that the *FVB-Met^M1248T/L1193V^* line was more permissive for tumor formation and on this background a dramatic shift in tumor type occurred from rare and infrequent carcinomas to highly aggressive mammary carcinomas [24]. As previously described [24], both nulliparous and multiparous *FVB-Met^M1248T/L1193V^* females developed aggressive mammary carcinomas on average by 11 months. Although other tumor types were identified, mammary carcinomas were the predominant tumor and present in 70% of female *FVB-Met^M1248T/L1193V^* mice. Expecting to see similar changes in tumor frequency and type, we created congenic strains on the FVB background for the other three *Met* alleles (D1226N; Y1228C; and M1248T). Nulliparous and multiparous females heterozygous for each knock-in mutation were aged until tumor growth required euthanasia. Morbidity was observed in several animals unrelated to tumor growth as detailed below.

**Figure 1:**
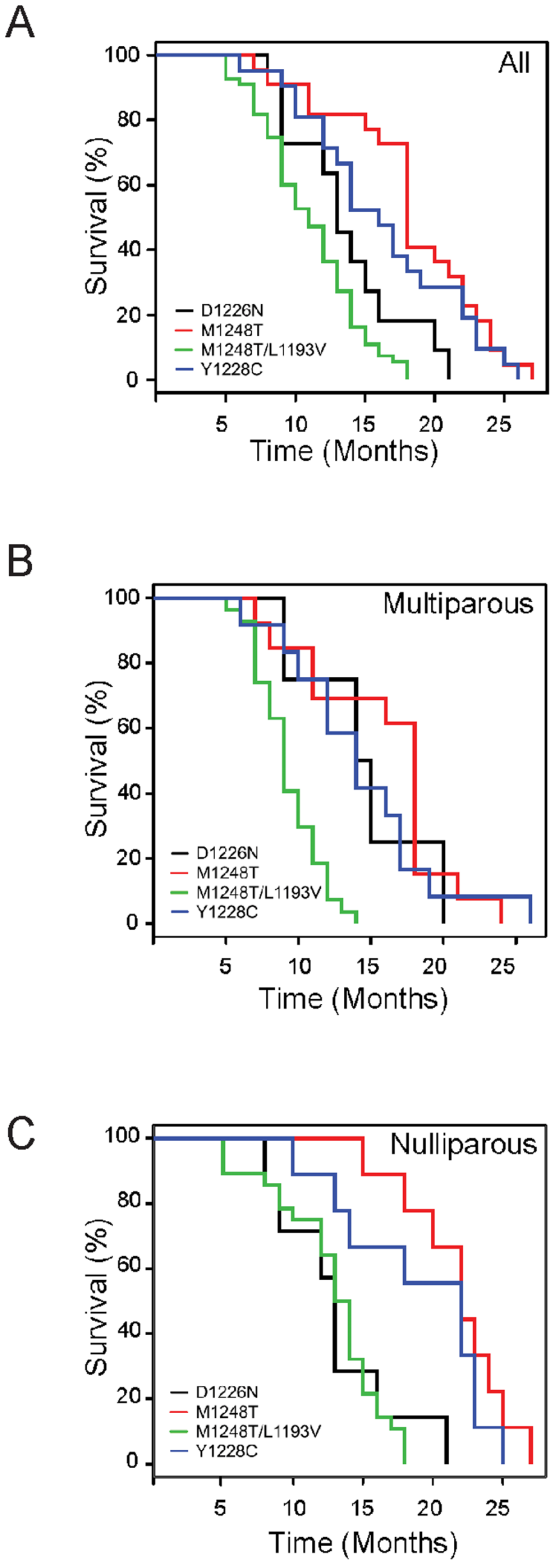
Survival analysis of *Met* mutant mice. Kaplan-Meier survival curves of A) all female animals, B) multiparous (>1 litter) females, and C) nulliparous females.

The *FVB-Met^M1248T/L1193V^* line developed tumors more rapidly than the three other mutant lines (Figure 1 and Table 1). The average survival age in the *FVB-Met^M1248T/L1193V^* mice was 11.0 months; the average age in the other three lines ranged from 12.6 to 18.3 months (differences with *FVB-Met^Y1228C^* (*p* = 0.005) and *FVB-Met^M1248T^* (*p*<3×10^−6^) were statistically significant). Parity decreased the time of tumor onset in the *FVB-Met^M1248T/L1193V^* mice but had a less predictable effect in the other lines (Figure 1). Multiparous females in the *FVB-Met^M1248T^* line had a shorter survival (15.8 months) than the *FVB-Met^M1248T^* nulliparous females (21.8 months), yet in the *FVB-Met^Y1228C^* and *FVB-Met^D1226N^* lines, multiparous females survived slightly longer than the nulliparous females. The differences between the lines are likely due to the predominant tumor phenotypes described below.

**Table 1:** Aging data for nulliparous and multiparous females for FVB-Met mutant lines.

	M1248T/L1193V	M1248T	Y1228C	D1226N
Total	56*	22	21	19
Nulliparous	28	9	9	12
Multiparous	27	13	12	7
Avg Age (months)	11.0	18.3	16.3	12.6
Multiparous Average Age (months)	9.3	15.8	18.9	12.8
Nulliparous Average Age (months)	12.8	21.8	14.3	11.9

Average age represents average time to death in months. *The parity of one *FVB-Met^M1248T/L1193V^* female was not recorded and this mouse was only included in the overall age statistics.

### Mutation-specific profiles are generated on the FVB background

**Figure 2:**
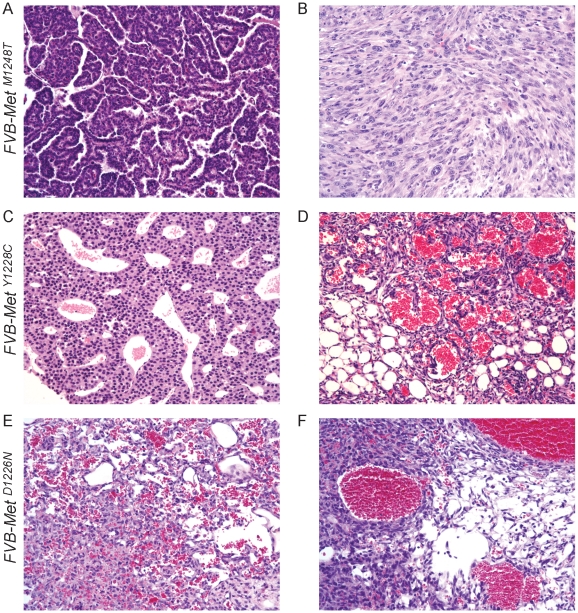
Spectrum of tumors observed in Met mutants. A) A bronchial alveolar adenocarcinoma and B) a histiocytic sarcoma in a *FVB-Met^M1248T^* mouse. C) A plasmacytoma and D) a sarcoma in a *FVB-Met^Y1228C^* mouse. E and F) Hemangiosarcomas observed in the mammary pads of two individual *FVB-Met^D1226N^* mice. All H&E images were taken at 200× magnification.

The tumor profiles among the four mutant FVB lines were strikingly different (Table 2). Carcinomas were prevalent in *FVB-Met^M1248T/L1193V^* mice; however, *B6-Met^M1248T/L1193V^* mice did not develop epithelia neoplasias in any tissue aside from a single occurrence of lung adenoma. Mammary tumors were the predominant cause of death in *FVB-Met^M1248T/L1193V^* mice; however, several animals also developed sarcomas or lymphomas. Once mammary tumors were identified, subsequent tumor growth was rapid in the *FVB-Met^M1248T/L1193V^* mice, especially in the multiparous females. This rapid growth likely resulted in the survival differences observed between the *FVB-Met^M1248T/L1193V^* mice and the other mutant lines (Figure 1). Only 18% of the *FVB-Met^M1248T^* mice developed mammary adenocarcinomas, whereas four animals developed bronchial alveolar adenocarcinomas and lymphomas, including histiocytic sarcomas (Table 2 and Figure 2A–B). Uterine leiomyoma and endometrial hyperplasia were rare observations. In the *FVB-Met^M1248T^* mice, sarcomas were also rare (1 out of 22 animals), which is consistent with the lack of sarcomas observed in *B6-Met^M1248T^* mice [19]. In the *FVB-Met^Y1228C^* mice, 24% developed mammary adenocarcinomas, but only one sarcoma and two lymphomas were observed (Table 2 and Figure 2C–D). The *FVB-Met^Y1228C^* line is the only other line to develop endometrial hyperplasia similar to that in the *FVB-Met^M1248T^* mice. However, this was only observed in one animal, and larger numbers of mice are necessary to determine if this is a significant phenotype. In the *Met^D1226N^* line, hemangiosarcomas were the predominant phenotype and were observed in 33% of *FVB-Met^D1226N^* (mu/+ and mu/mu) mice and in 72% of *B6-Met^D1226N^* (mu/mu) mice [19]. This is the only tumor type for any of the four knock-in mutations that was consistently observed on both backgrounds. Surprisingly, the majority of the hemangiosarcomas in the *Met^D1226N^* mice were identified in the mammary pad (Table 2 and Figure 2E–F); this is an uncommon location for hemangiosarcomas in mice. Only one mammary adenocarcinoma was observed in this line. In each of the lines, bronchial alveolar adenocarcinomas were identified. However, it is not clear whether this is specifically associated with the Met mutations as this is a relatively common neoplasm observed in wild-type FVB animals (observed in 18% of FVB/N females by 24 months) [25].

**Table 2:** Histopathology of Met mutant lines on the FVB and B6 backgrounds.

	M1248T/L1193V		M1248T		Y1228C		D1226N	
	FVB	B6	FVB	B6	FVB	B6	FVB	B6
Carcinomas								
Mammary adenocarcinoma	39	–	4	–	5	–	1	–
Salivary gland squamous carcinoma	–	–	–	–	–	–	1	–
Brochial alveolar adenocarcinoma	1	–	3	1	2	–	1	–
Transitional cell carcinoma	–	–	–	1	–	–	–	–
Squamous cell carcinoma	–	–	–	1	–	–	–	–
Hemangiosarcoma	5	4	1	–	–	10	5	13
Sarcomas**	3	3	–	–	1	2	1	2
Lymphomas*	1	2	2	7	2	6	–	–
Uterine leiomyoma	–	–	1	–	–	–	–	–
Endometrial hyperplasia	–	–	1	–	1	–	–	–
Nonremarkable#	9	3	9	1	6	–	9	–
**Total Mice**	56	18	22	17	21	19	19	18

Mammary adenocarcinomas were found with a significantly higher proportion in *FVB-Met^M1248T/L1193V^* compared to *B6-Met^M1248T/L1193V^* mice (*p* = 2.4×10^−6^). Hemangiosarcomas developed at a higher incidence in *B6-Met^Y1228C^* and *B6-Met^D1226N^* mice compared to the FVB lines (Y1228C, *p* = .0005; D1226N, *p* = .01). *Lymphomas included plasmacytomas and histiocytic sarcomas on the FVB background and lymphoblastic, lymphocytic, histiocytic sarcomas, and follicular center lymphomas on the B6 background. **Sarcomas included a rhabdomyosarcoma and undifferentiated sarcomas on the FVB background and fibrosarcomas, myxomas, leiomyosarcomas, and spindle-like sarcomas on the B6 background.

# Animals without any identifiable tumor burden or cause of death are referred to as “nonremarkable”.

Several mice in each line were euthanized due to signs of morbidity that were not associated with any signs of tumor development; these included several animals that displayed enlarged abdomens with increased abdominal fat. Several animals were also euthanized due to hypoactivity and pathologic evaluation did not reveal cause of morbidity. A few mice had cellular infiltrates in multiple organs consistent with bacterial infection. These animals are designated as “Nonremarkable” in Table 2.

### Distinct mammary phenotypes are observed in Met mutant lines

**Figure 3:**
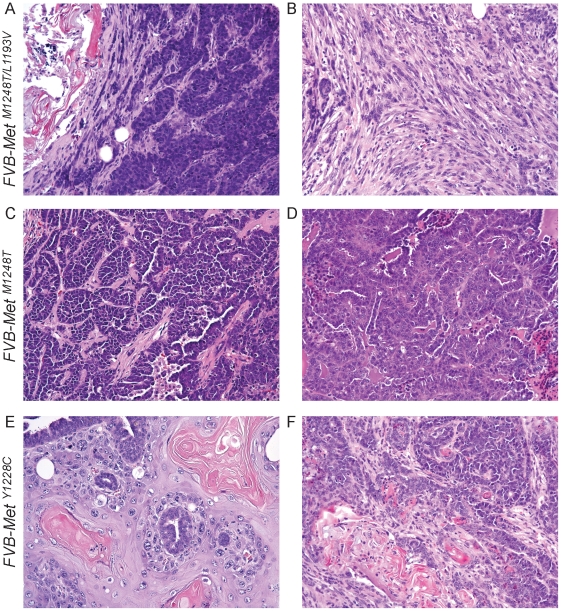
Mammary tumor histology observed in Met mutants. A) Adenocarcinoma with solid patterns and squamous metaplasia in a *FVB-Met^M1248T/L1193V^* mouse; B) myoepithelioma in a *FVB-Met^M1248T/L1193V^* mouse; C and D) adenocarcinomas with tubular patterns from two individual *FVB-Met^M1248T^* mice; E) squamous cell carcinoma observed in a *FVB-Met^Y1228C^* mouse; and F) an adenosquamous carcinoma observed in a *FVB-Met^Y1228C^* mouse. All H&E images were taken at 200× magnification.

**Figure 4:**
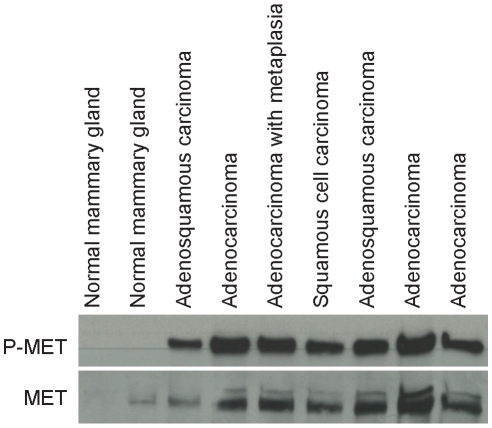
Met activation and expression is increased in *FVB-Met^M1248T/L1193V^* mammary tumors. Western blot analysis of immunoprecipiated lysates showed increased Met activation (phospho-Met) and expression in mammary tumors isolated from *FVB-Met^M1248T/L1193V^* mice compared to normal *FVB-Met^M1248T/L1193V^* mammary pads. Met activation and expression was increased in all of the pathologic types observed in the *FVB-Met^M1248T/L1193V^* mammary tumors.

**Figure 5:**
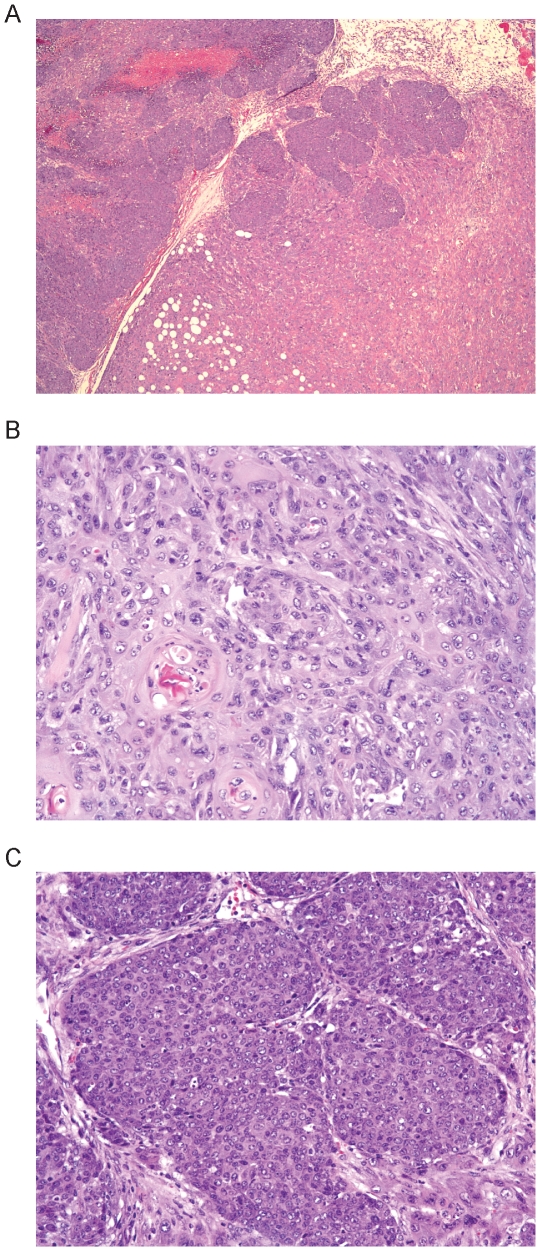
Contiguous tumors observed in a *Met^M1248T/L1193V^* mouse. A) Two adjacent mammary tumors within a *FVB-Met^M1248T/L1193V^* mouse (40× magnification). B) Adenocarcinoma shown in A with squamous metaplasia and fibromatous changes (200× magnification). C) Adenocarcinoma shown in A with solid patterns (200× magnification).

Because we observed distinct tumor profiles among the Met mutant lines on the B6 background, the mutation-specific differences we observed on the FVB background were not unexpected. The occurrence of diverse tumor patterns on two backgrounds confirmed that the germline Met mutations induce tumorigenesis in multiple cell types. However, there appeared to be mutation-specific variation among the mammary tumor histopathology. As previously mentioned, the *FVB-Met^M1248T/L1193V^* mice had the most penetrant mammary tumorigenic phenotype (Table 2). In addition, the mammary tumors that developed in *FVB-Met^M1248T/L1193V^* females had a wide range of pathological diversity [24], including squamous metaplasia, solid and tubular patterns, and myoepitheliomas (Figure 3A–B). In contrast, *FVB-Met^M1248T^* mammary tumors displayed only tubular and acinar patterns without any observed squamous metaplasia (Figure 3C–D). The histological appearance of *FVB-Met^Y1228C^* mammary tumors was more consistent with that of the *FVB-Met^M1248T/L1193V^* phenotype, where 60% of *FVB-Met^Y1228C^* mammary tumors contained significant squamous metaplasia and 40% displayed solid and tubular patterns (Figure 3E–F). We also observed mammary hyperplasia in 2 of 21 *FVB-Met^Y1228C^* mice. Increased Met activation and expression was present in each of the *FVB-Met^M1248T/L1193V^* mammary tumor types (Figure 4). All tumors found in the *FVB-Met^M1248T^*, *FVB-Met^Y1228C^, and FVB-Met^D1226N^* mice were unifocal (62 total animals) whereas multifocal tumors were identified in 6 *FVB-Met^M1248T/L1193V^* females (of 56 total) characterized. Although multifocal mammary tumors are not unique in mouse models, the fact that the multifocal *FVB-Met^M1248T/L1193V^* tumors had distinct morphologic patterns is uncommon. The most remarkable case was a *FVB-Met^M1248T/L1193V^* mouse that developed two contiguous tumors with distinctive morphologic characteristics in the mammary pad (Figure 5A). These tumors had distinct, closely opposed borders consistent with expansile growth of both tumors. These contiguous tumors included an adenocarcinoma with squamous metaplasia and fibromatous changes (Figure 5B) and an adenocarcinoma with solid patterns (Figure 5C). These distinctive tumors, along with the other *FVB-Met^M1248T/L1193V^* mice that developed multiple distinctive mammary tumors, suggests that mutationally activated Met is being expressed in a progenitor population leading to diverse mammary pathology.

## Discussion

Previously we have shown that activating knock-in mutations in the Met kinase domain unexpectedly gave rise to mutation-specific tumor profiles on the B6 background. For example, *B6-Met^M1248T^* mice develop carcinomas, whereas *B6-Met^D1226N^* mice primary developed hemangiosarcomas. Here we asked two questions: when expressed on a different background (FVB), will activating Met mutations induce the same tumor type specificity? And what role, if any, does the host background play?

When knock-in activating alleles were placed on the FVB background, tumor latency significantly decreased and a more highly penetrant carcinoma phenotype was generated. Since this is a knock-in mouse model, each mutation is expressed under the endogenous promoter, and therefore phenotypic differences were not due to differences in insertion sites or differential expression levels. The variation in tumor profiles observed on both the B6 and FVB backgrounds is markedly comparable to the diverse allele-specific spectra observed in p53-deficient mouse models of Li-Fraumeni syndrome [26]. The striking phenotypic differences observed for each mutation may be due to differential genetic interactions with modifying alleles or cell-type dependent signaling interactions (i.e. endothelial vs. epithelial).

Several of the tumor-specific phenotypes were consistent between the B6 and FVB backgrounds. For instance, the *Met^D1226N^* mice developed hemangiosarcomas at a high frequency on both backgrounds, whereas the *Met^M1248T^* mice rarely developed sarcomas on either. On the other hand, we also observed distinct tumor-specific phenotypes between the two backgrounds. Mammary adenocarcinomas were the most common neoplasia in all FVB lines, whereas mammary tumors were not observed on the B6 background. This may be in part due to the development of parity-associated hyperplasia in FVB females [27], though mammary adenocarcinomas were also observed in virgin animals. Mammary adenocarcinomas were most frequent and had the shortest time of onset in the *FVB-Met^M1248T/L1193V^* line, which is not unexpected considering that the *Met^M1248T/L1193V^* double mutant is more tumorigenic in *in vitro* and *in vivo* assays [20].

MET overexpression is observed in 20–30% of human breast cancers and is a strong, independent predictor of decreased survival [28], [29], [30]. In the current study, we identified allele-specific pathological phenotypes of several Met mutations. *FVB-Met^M1248T/L1193V^* and *FVB-Met^Y1228C^* mice developed similar mammary tumors with diverse features such as squamous metaplasia and solid and tubular patterns. On the other hand, *FVB-Met^M1248T^* mammary tumors only displayed acinar and tubular features, but squamous metaplasia was not observed. These results indicate that activation of Met is able to initiate neoplasia in multiple mammary cell types and that Met mutations may uniquely influence cellular differentiation in the mammary gland during tumorigenic growth.

It is noteworthy that only one copy of a mutated *Met* allele (expressed under the endogenous promoter) is able to initiate such diverse tumor phenotypes. This underscores the affect that even a modest increase in Met activity may have on cell transformation. Amplification of the *MET* allele has been observed in gastric and lung cancers [15], [31], [32]. On the other hand, trisomy of chromosome 7 (containing the *MET* locus) is sufficient for papillary renal carcinomas [33]. These observations in human tumors, in addition to our work in mouse models, suggest that even minor amplification of the *MET* allele may have significant consequences in various cell types. Therefore, future cytogenetic analysis should evaluate cancers for both high and low levels of *MET* amplification.

We show that Met is able to initiate tumorigenesis in multiple cell types—including epithelial, hematopoietic, and endothelial—on two distinct mouse backgrounds. The mutation-specific spectra observed on the FVB strain validate our unique mutation-specific findings on the B6 background. These results indicate that each mutation has distinct effects on downstream signaling that may be influenced by the cellular environment and/or modifying genes. Furthermore, these results support the numerous studies in human cell lines and tissues that demonstrate aberrant MET activity is involved in the initiation, progression, and metastasis of diverse cancers (www.vai.org/metandcancer). More recently, *MET* amplification and activation via HGF/SF has also been implicated in resistance to EGFR inhibition in lung cancer [31], [34]. The fact that Met influences tumorigenesis from initiation to metastasis in numerous cell types makes the Met-HGF/SF pathway a critical therapeutic target.

Here we describe a preclinical model in which Met and/or HGF/SF inhibition can be assessed in multiple tumor types. Further studies are required to determine which genetic modifiers influence the tumorigenic phenotype of an activating Met mutation. Met mutations have been identified in a wide range of tumors including renal carcinomas, mesotheliomas, gastric carcinomas, and non-small cell and small cell lung cancers. Therefore, it is imperative that we advance our understanding of which factors genetically influence or interact with Met. Greater insight into the interaction of genetic modifiers of Met signaling will significantly impact on our ability to tailor combination therapies for Met-driven cancers.

## Materials and Methods

### Animals

Germline knock-in lines were derived on a mixed C57BL/6 background as previously described [19]. Congenic FVB/N animals were derived by speed congenic MAX-BAX^SM^ backcrossing (Charles River Laboratories). Animals were housed in the VAI Vivarium. Housing and care of the animals were in strict accordance with the guidelines established by the VAI Institutional Animal Care and Use Committee. All research involving animals was approved by the VAI Institutional Animal Care and Use Committee (Permit Numbers: 05-08-023 and 08-10-029).

### Tumor analysis

Mice were examined biweekly for tumor development and were euthanized when tumors were between 1–2 cm^3^. Tumor samples along with selected organs were surgically isolated and fixed in 4% paraformaldehyde/phosphate-buffered saline for 24 h. Fixed tissues were dehydrated, paraffin-embedded, cut into 5-µm sections, and stained with hematoxylin and eosin (H&E).

### Immunoprecipitation and Western Blot Analyses

Tissue was homogenized in SBN lysis buffer [50 mM Tris (pH 7.5), 150 mM NaCl, 10% glycerol, 1% Nonidet P-40, 1 mM EGTA], 1 mM sodium orthovanadate, and Complete Proteinase Inhibitor Mixture Tablets (Roche Applied Science). For immunoprecipitation, 0.5 mg of protein was incubated overnight with anti-Met antibody (B-2, Santa Cruz Biotechnology) at 4°C. Immune complexes were collected with protein A-Sepharose beads (RepliGen) and washed three times in HNTG buffer [20 mM HEPES (pH 7.5), 150 mL NaCl, 0.1% TritonX-100, 10% glycerol]. Immunoprecipitated lysates were separated on a 4–20% Tris-glycine gel (Invitrogen), transferred to a nitrocellulose membrane (Invitrogen), and examined by Western analysis using a phospho-Met (Tyr1234/1235) antibody (Clone 3D7, Cell Signaling) and an anti-Met antibody (B-2).

### Statistical Analysis

Survival was determined using the Kaplan and Meier survival function. Pairwise comparisons of survival curves were done using a log-rank test. To compare differences in the proportion of tumor types in each background, a two proportion z test for equal proportions was used. Proportions were considered significantly different if the *p*-value <0.05.
